# DGDRP: drug-specific gene selection for drug response prediction via re-ranking through propagating and learning biological network

**DOI:** 10.3389/fgene.2024.1441558

**Published:** 2024-09-20

**Authors:** Minwoo Pak, Dongmin Bang, Inyoung Sung, Sun Kim, Sunho Lee

**Affiliations:** ^1^ Department of Computer Science and Engineering, Seoul National University, Seoul, Republic of Korea; ^2^ Interdisciplinary Program in Bioinformatics, Seoul National University, Seoul, Republic of Korea; ^3^ Aigendrug Co., Ltd., Seoul, Republic of Korea; ^4^ Interdisciplinary Program in Artificial Intelligence, Seoul National University, Seoul, Republic of Korea

**Keywords:** drug response, gene ranking, gene selection, network propagation, graph neural network, biological network

## Abstract

**Introduction:** Drug response prediction, especially in terms of cell viability prediction, is a well-studied research problem with significant implications for personalized medicine. It enables the identification of the most effective drugs based on individual genetic profiles, aids in selecting potential drug candidates, and helps identify biomarkers that predict drug efficacy and toxicity.A deeper investigation on drug response prediction reveals that drugs exert their effects by targeting specific proteins, which in turn perturb related genes in cascading ways. This perturbation affects cellular pathways and regulatory networks, ultimately influencing the cellular response to the drug. Identifying which genes are perturbed and how they interact can provide critical insights into the mechanisms of drug action. Hence, the problem of predicting drug response can be framed as a dual problem involving both the prediction of drug efficacy and the selection of drug-specific genes. Identifying these drug-specific genes (biomarkers) is crucial because they serve as indicators of how the drug will affect the biological system, thereby facilitating both drug response prediction and biomarker discovery.**Methods:** In this study, we propose DGDRP (Drug-specific Gene selection for Drug Response Prediction), a graph neural network (GNN)-based model that uses a novel rank-and-re-rank process for drug-specific gene selection. DGDRP first ranks genes using a pathway knowledge-enhanced network propagation algorithm based on drug target information, ensuring biological relevance. It then re-ranks genes based on the similarity between gene and drug target embeddings learned from the GNN, incorporating semantic relationships. Thus, our model adaptively learns to select drug mechanism-associated genes that contribute to drug response prediction. This integrated approach not only improves drug response predictions compared to other gene selection methods but also allows for effective biomarker discovery.**Discussion:** As a result, our approach demonstrates improved drug response predictions compared to other gene selection methods and demonstrates comparability with state-of-the-art deep learning models. Case studies further support our method by showing alignment of selected gene sets with the mechanisms of action of input drugs.**Conclusion:** Overall, DGDRP represents a deep learning based re-ranking strategy, offering a robust gene selection framework for more accurate drug response prediction. The source code for DGDRP can be found at: https://github.com/minwoopak/heteronet.

## 1 Introduction

As the paradigm of drug treatment shifts from a “one-size-fits-all” approach to personalized medicine, drug response prediction has become an essential task. Drug response prediction, especially in terms of cell viability prediction, is a well-studied research problem with significant implications for personalized medicine. It enables the identification of the most effective drugs based on individual genetic profiles, aids in selecting potential drug candidates, and helps identify biomarkers that predict drug efficacy and toxicity. Although some methodologies integrate multi-omics data for drug response prediction (Sharifi-Noghabi et al., 2019; Oh et al., 2020; Feng et al., 2021), many researchers prefer to focus solely on transcriptomic data due to its higher availability, relatively lower cost, and the critical role of gene expression in reflecting cellular states and responses (Jiang et al., 2022; Shin et al., 2022; Yang and Li, 2023; Bang et al., 2024).

A deeper investigation into drug response prediction reveals that drugs exert their effects by targeting specific proteins, which subsequently perturb related genes in cascading ways. This perturbation influences cellular pathways and regulatory networks, ultimately affecting the cellular response to the drug. Identifying which genes are perturbed and understanding their interactions can provide critical insights into the mechanisms of drug action. Therefore, the problem of predicting drug response can be framed as a dual problem involving both the prediction of drug efficacy and the identification of drug-specific genes. Recognizing these drug-specific genes (biomarkers) among omics data is crucial because they serve as indicators of how the drug will affect the biological system, thereby facilitating both accurate drug response prediction and biomarker discovery.

Despite the availability of extensive omics data, effectively utilizing it for drug response prediction poses significant computational challenges. One primary issue is the high-dimensionality, low-sample problem, characterized by a substantial imbalance between the number of gene features and the available samples. While sequencing technologies allow for the measurement of various biological entities, including RNA, DNA, proteins, and metabolites, obtaining trainable patient samples involves legal and ethical challenges. This imbalance often leads to overfitting, where models perform well on training data but fail to generalize to new, unseen data (Adam et al., 2020).

The dual approach is crucial because gene selection methods not only reduce the dimensionality of omics data but also facilitate the discovery of biomarkers that are directly linked to drug response. Despite numerous biomarker identification methods, only a few specifically target drug response prediction. The problem of drug response prediction can thus be formulated as:

$$\text{Biomarkers}=q_{\phi }\left(\text{Drug},\text{Target}\right)$$

$$\text{Drug Response}=p_{\theta }\left(\text{Drug},\text{Profile of identified biomarkers}\right)$$

where the predictive function $q_{\phi }$ identifies the relevant biomarker genes based on the drug, its target, and the biological network, and $p_{\theta }$ predicts the drug response using the profile of these biomarkers along with the drug information.

Various methods have been developed for gene selection to reduce the dimensionality of omics data and facilitate biomarker discovery. These methods generally fall into four categories; the first is fixed sets, where knowledge-guided predefined gene sets such as the L1000 Landmark gene set (Subramanian et al., 2017), drug target genes, and cancer hallmark genes (Menyhárt et al., 2016) are utilized. These fixed sets serve as a starting point for identifying key biomarkers, although they may not account for the specific characteristics of different drugs or patient samples.

The second set is trainset-dependent sets, which include gene sets selected based on differential expression (DEG) or gene expression variance in the training dataset. These approaches, however, often lack the specificity and adaptability required for optimal gene selection.

ML-driven sets, the third category, filter genes using feature importance scores obtained from machine learning algorithms like logistic regression or random forest Ding et al. (2016). Although these methods are dynamic and data-driven, they often lack biological interpretability and may not be tailored to the specific mechanisms of individual drugs, limiting their effectiveness in discovering relevant biomarkers.

Lastly, knowledge-graph based ranking methods including network propagation perform ranking based on an input gene set and biological network knowledge to identify relevant genes (Cowen et al., 2017). Given the complexity of gene interactions, effective drug response prediction and gene selection must account for gene-gene interactions. Many existing studies have utilized network propagation techniques to model these complex interactions. However, these methods are often insufficient for identifying drug-target-based genes, and also lacking the context about the semantics between the ranked genes. For example, there is no information on how the genes ranked as first and second are associated with each other, limiting the understanding of gene interactions and their collective impact on drug response and biomarker discovery, thus necessitating the development of new computational strategies.

**FIGURE 1:**
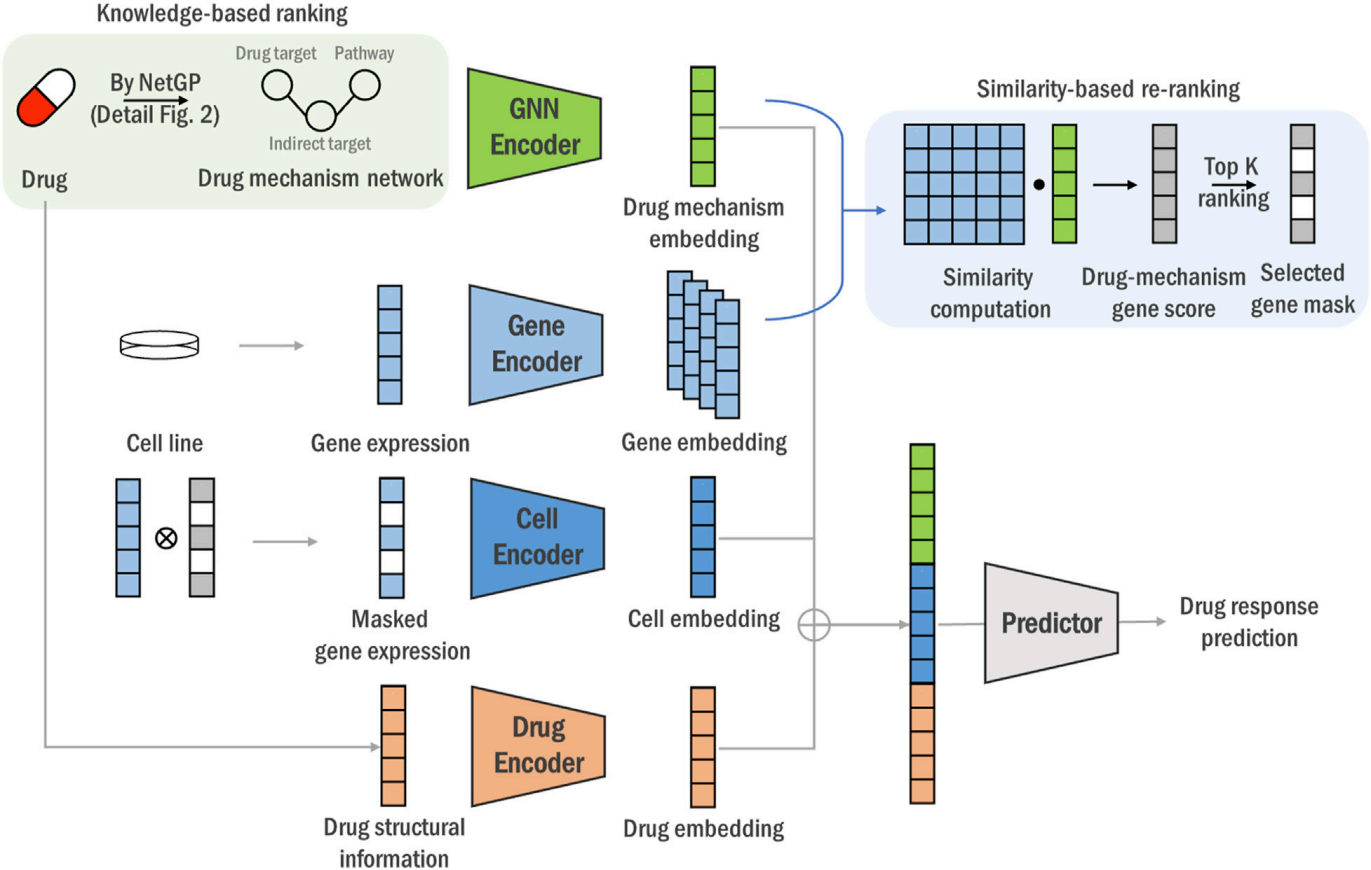
The overall end-to-end framework of the DGDRP. For each drug’s drug target information and protein-protein interaction network, we rank genes and obtain indirect targets through NetGP. And by incorporating pathway information, we construct a dru mechanism network (green box part, details is illustrated in Figure 2) Then, a GNN encoder for drug mechanism network and a gene encoder for cell line expression are utilized to generate drug mechanism and gene embedding vectors, respectively, and re-rank genes by calculating the similarity between drug target and gene embedding vector (blue box). Finally, we predict the drug response value using the expression values of the top-k genes, along with the drug mechanism and structural information.

To overcome these limitations, we propose DGDRP (Drug-specific Gene selection for Drug Response Prediction), a novel graph neural network (GNN)-based model designed for both knowledge-based and data-driven drug-specific gene selection (Figure 1. Our approach involves a unique rank-and-re-rank process that enhances the specificity and adaptability of gene selection, allowing for simultaneous drug-response biomarker identification and drug response prediction:

1. Knowledge-based ranking: We utilize drug target information to rank and select genes related to each drug’s mechanism through a pathway knowledge-enhanced network propagation algorithm, NetGP (Pak et al., 2023). This ensures that the selected genes are biologically relevant to the drug’s action. The top-k genes based on NetGP scores are used to construct the drug mechanism network, a heterogeneous network of drug-specific proteins and pathways, capturing the intricate relationships between drug-affected genes and their associated biological processes.
2. Similarity-based re-ranking: Genes are re-ranked based on the similarity between the gene embedding and drug target embedding vectors generated by through learning the drug mechanism network with GNN. This similarity-based ranking incorporates semantic information among the ranked genes, enabling the understanding of their interrelationships and relevance to the drug response.

Utilizing the re-ranked gene set, DGDRP selects the top-k genes and predicts drug response using the transcriptomic profile of the gene set along with drug structural information. As far as we are aware, no existing studies simultaneously address both drug response prediction and drug-specific gene selection.

The rationale behind our re-ranking strategy is rooted in the understanding that gene functions are influenced by their interactions with neighboring genes. Embedding representations of genes are created by considering information about their neighboring genes, which allows us to capture the intricate relationships within the gene network.

By integrating both knowledge-based and data-driven approaches, our method offers improved predictions of drug response compared to other gene selection methods and demonstrates comparability with state-of-the-art deep learning models as a stand-alone model. These results indicate that our approach successfully addresses the high-dimension, low-sample problem, enhancing the accuracy and reliability of computational drug response models. Case studies on the selected gene sets also demonstrate the alignment of the gene set-associated pathways with the mechanism of action (MoA) of the input drugs.

## 2 Materials and methods

### 2.1 Dataset

In this study, we formulated the problem of drug response prediction an inference task that predicts drug response value given cell line gene expression profile along with treated drug’s structure and target information. The drug response end point used in this study is the half maximal inhibitory concentration (IC50) that indicates the concentration of a drug that is required to inhibit a biological function or process by half, in this case, cell viability. Since IC50 takes on the form of a continuous value, the drug response prediction task can be formulated as a regression task. The data for cell line is represented by transcriptomic gene expression profile, and the data for drug is represented by Simplified Molecular Input Line Entry System (SMILES) sequence.

In this study, we utilized the GDSC (Yang et al., 2012) (https://www.cancerrxgene.org) and NCI-60 databases (Shoemaker, 2006) as the primary source for drug response information. As one of the most comprehensive resources for drug response data, GDSC contains 576,758 dose-response curves. It comprises two versions. GDSC1, which includes 970 cell lines, 403 drugs and 333,292 IC50 values, and GDSC2, which consists of 969 cell lines, 297 drugs and 243,466 IC50 values. We used data from both versions in this study, using the values from GDSC2 when there were overlaps in drug response information. NCI-60 database includes the measured GI50 (growth inhibition 50) values with smaller set of total 59 cell lines, 215 drugs and 12,685 GI50 values.

We acquired the SMILES data of the drugs from the GDSC, NCI-60 and CADD Group Chemoinformatics Tools and User Services (https://cactus.nci.nih.gov/). Using the RDKit Python package (https://www.rdkit.org), we canonicalized the SMILES strings and generated Morgan fingerprints that were then fed into the models requiring fingerprint properties of drugs as input. Regarding cell line gene expression data, we initially obtained profiles of 18,115 genes for GDSC and 18,077 genes for NCI-60. However, due to their substantial memory and computation resource requirements, we conducted preliminary filtering to eliminate genes with minimal expression variation, resulting in profiles of 10,000 genes. We further utilized the GDSC and DrugBank (Wishart et al., 2018) databases to obtain the drug-target information.

The biological network was obtained from the STRING database v11.5 (https://string-db.org/, accessed July 2023). In the STRING Protein-Protein Interaction (PPI) network, each node represents a protein, while each edge indicates the interaction between two proteins. These interactions can be both direct (physical) or indirect (functional), supported by evidence from computational prediction, text mining, and laboratory experiments, including co-immunoprecipitation and yeast two-hybrid system (Hu et al., 2021; Szklarczyk et al., 2021; Bultinck et al., 2012).

The STRING dataset also offers the score of each edges, and allows the user to select the desired confidence level of the network. Among various pre-defined thresholds of 0.9 (high confidence), 0.7 (high confidence), 0.4 (medium confidence) and 0.15 (low confidence), we utilized 0.8 and 0.9 for GDSC and NCI-60 respectively, during the knowledge-based ranking via NetGP algorithm. Further more, during the drug mechanism network construction, we did not utillize any cutoff value and utilized all edges provided by the STRING database.

While STRING was the primary source for constructing our PPI networks, we also explored alternative datasets such as HitPredict for performance comparisons. HitPredict is another PPI database that integrates experimentally validated physical interactions (Patil et al., 2011). Although not utilized in the final model, these comparisons are documented in the Supplementary Material to provide a broader context for our network selection choices.

For the purpose of training and evaluation, we only incorporated drugs with complete SMILES, drug response, and target information in the each databases. Drugs missing any of these data points were excluded. Furthermore, drugs with target proteins not present on the STRING PPI network were also omitted. Following this filtration process, the final drug response dataset used in this study consisted of 227 drugs, 804 cell lines, and 168,244 drug response values for GDSC database, and 118 drugs, 59 cell lines, and 6,962 drug response values for NCI-60 database.

### 2.2 Model structure

This study introduces a deep learning model, DGDRP, which selectively chooses genes in a drug-specific manner through a rank-and-re-rank process. The structure of DGDRP is illustrated in Figure 1. The model comprises two main parts: the gene-selection step and the drug response prediction step.

The upper part of Figure 1 shows the gene-selection process, which uses embeddings of the biological drug mechanism derived from drug target information, cell line gene expression profiles, and protein-protein interaction (PPI) networks. The lower part of Figure 1 illustrates the prediction of drug response, based on the combined embeddings of the drug mechanism, drug chemical properties, and cell line gene expression profiles.

In the rank-and-re-rank gene-selection step, a heterogeneous network is constructed for each drug, incorporating connections between drug targets and related pathways. Initially, genes are ranked based on the network propagation, which represents the systemic propagation of biological mechanism of the drug. The top-ranked genes, namely, ‘indirect targets’, is then integrated with direct target genes ans pathway information, constructing a knowledge graph that provides a comprehensive view of the drug’s biological impact.

Subsequently, a re-ranking process is performed where genes are re-evaluated based on the similarity between the cell line embeddings and the refined network embeddings obtained from a Graph Neural Network (GNN). This re-ranking step ensures that the selected genes are contextually related, offering a deeper insight into their interactions and relevance to the drug mechanism.

By integrating the rank-and-re-rank gene-selection process into the learning model, the entire procedure is performed in an end-to-end manner. This approach enhances the specificity and adaptability of gene selection, improving the accuracy of drug response predictions. The following sections provide detailed descriptions of each steps on the network propagation-based ranking, heterogeneous network construction and drug response prediction.

### 2.3 Rank-and-re-rank gene selection

#### 2.3.1 Knowledge guided propagation-based ranking (NetGP)

DGDRP employs a unique method for gene selection, leveraging a knowledge-enhanced network propagation algorithm, NetGP (Pak et al., 2023). The core of NetGP is the network propagation algorithm Cowen et al. (2017), which performs propagation of gene effects throughout the network. This algorithm is fundamentally associated with the Random Walk with Restart (RWR) technique, where the probability distribution vector $p_{t}$ at step t is updated iteratively until convergence as follows:

$$p_{t+1}=\left(1-\alpha \right)Wp_{t}+\alpha p_{0}$$

Here, W is the column-normalized adjacency matrix of the network, α is the restart probability, and $p_{0}$ is the initial probability vector indicating the starting positions or ‘seed genes’ (e.g., drug targets). The network propagation can be viewed as a simulation technique that computes the effect of drug-target interactions throughout the cell, enabling quantification of the degree of perturbations for each gene resulting from drug treatment with the consideration of gene interactions.

NetGP enhances this simulation by reinforcing the ranking process with pathway knowledge during propagation. Specifically, it incorporates a gene set enrichment algorithm to adjust the propagation dynamically, ensuring that the influence of pathway-relevant genes is amplified. This integration of pathway knowledge allows NetGP to provide a more accurate and contextually relevant ranking of genes, reflecting the biological mechanisms of drug action more effectively. Since our model leverages the STRING PPI network to identify and rank genes, it is essential that the same gene set is used as the background in the enrichment analysis to ensure that the results are relevant and accurately reflect the biological context of the STRING network.

With each drugs’ ‘direct target’ genes as seeds, we performed NetGP algorithm and obtained the NetGP propagation scores for each gene on the network. Then, we defined the top 20 genes with the highest scores as ‘indirect targets’. High NetGP scores imply that the corresponding genes are significantly perturbed by the drug. Since a drug acts by perturbing the target proteins and the perturbations propagate through protein-protein interactions, we regarded the most significantly perturbed genes as indirect targets.

#### 2.3.2 Drug mechanism network construction

After obtaining the direct and indirect targets, we then constructed a heterogeneous drug mechanism network by connecting the targets with biological pathways that contain them. The intra-target connections between direct targets and indirect targets are determined by the STRING database (Szklarczyk et al., 2021). As mentioned above, we did not apply any filtering criterion and utilized all the edges from the database. Additionally, the associations between the indirect target genes and the pathways are established as defined by the KEGG database (Kanehisa and Goto, 2000). This process yields one representative network per drug, containing its mechanism-relevant genes and pathways, which is then fed to a single overviewing graph neural network that is trained on all the drug mechanism networks.

**FIGURE 2:**
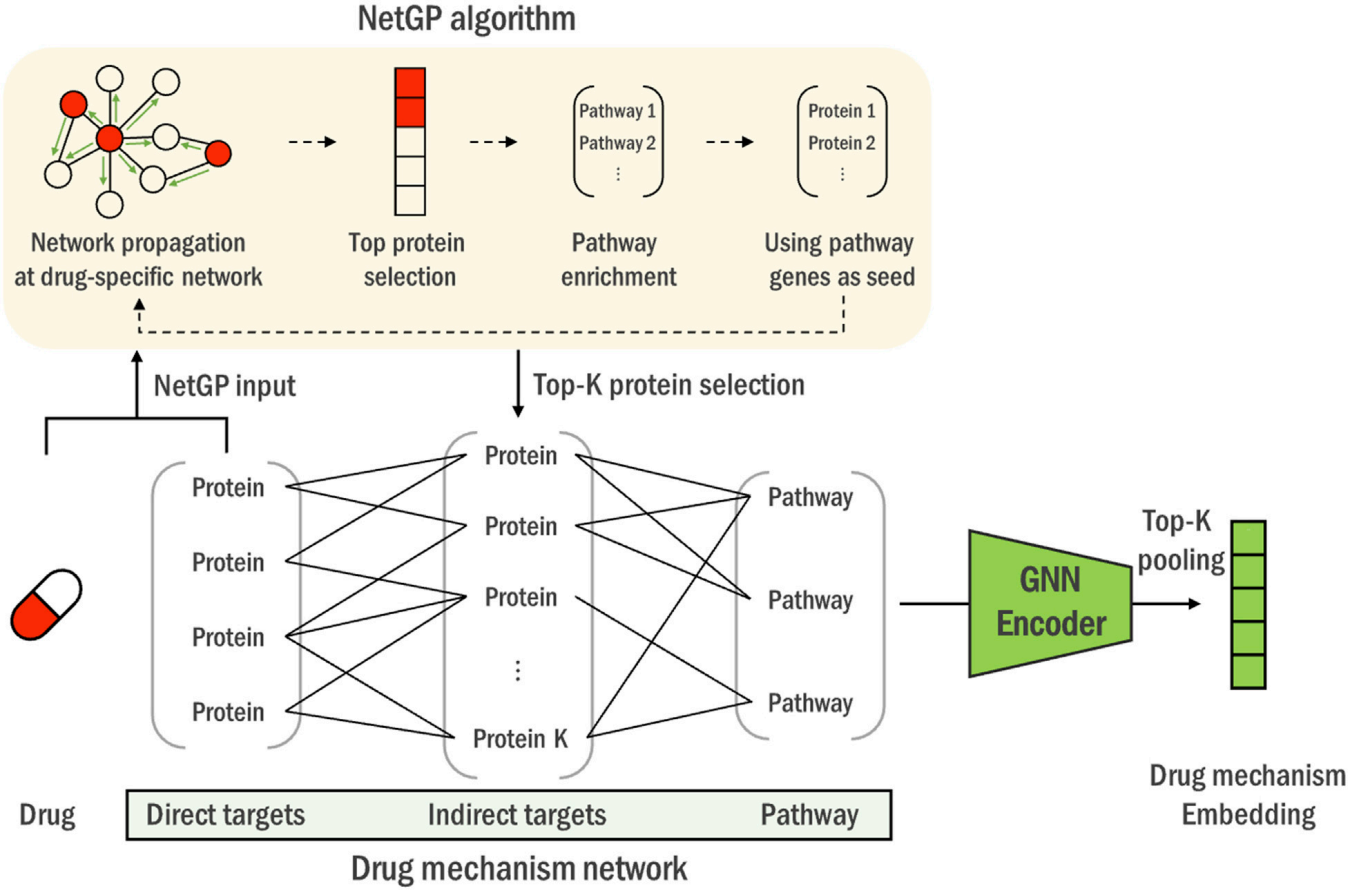
The construction process of the heterogeneous drug mechanism network. Using drugs’ direct targets as seeds, NetGP algorithm is performed on the PPI network (yellow box). Genes are then ranked based on their NetGP scores to identify the top K indirect targets. Lastly, pathways associated with indirect targets are merged to construct a heterogeneous graph of direct, indirect targets and pathway entities. Drug mechanism networks of all the drugs are then encoded through a universal graph neural network into a drug mechanism embedding vector.

The heterogeneous drug mechanism network G=(V,E) , with V and E as its nodes and edges, is illustrated in Figure 2 and can be formulated as follows:

$$\begin{array}{lll} & <Heterogeneous\;Graph & >\\ T_{D} & =\left\{g_{1},g_{2},\ldots ,g_{d}\right\}:\text{a set of direct target genes} & \\ T_{I} & =\left\{g_{1},g_{2},\ldots ,g_{i}\right\}:\text{a set of indirect target genes} & \\ G_{T} & =\left(V_{T},E_{T}\right):\text{graph connecting }T_{D},T_{I} & \\ P_{I} & =\left\{p_{1},p_{2},\ldots ,p_{k}\right\}:\text{a set of pathways containing }T_{I} & \\ G_{P} & =\left(V_{P},E_{P}\right):\text{graph connecting }T_{I},P_{I} & \\ G & =\left(V_{T}+V_{P},E_{T}+E_{P}\right):\text{heterogeneous graph} & \end{array}$$

where d and i are the number of direct target genes and indirect target genes respectively. $V_{T}$ denotes the set of combined genes of $T_{D}$ and $T_{I}$ . $E_{T}$ denotes the set of gene-gene interactions between $T_{D}$ and $T_{I}$ . k is the number of KEGG pathways that contains $T_{I}$ .

The resulting heterogeneous network, which captures both direct and contextual biological interactions relevant to each drug, enables the quantification of complex gene-pathway relationships and is further learned through a graph neural network to represent the drug-target information into an embedding vector suitable for drug response prediction.

#### 2.3.3 Deep learning and embedding similarity-based re-ranking

The generalizability of deep learning roots in its ability to generate expressive embedding space. Using the drug-specific heterogeneous network constructed in the previous step, gene selection is performed using the similarity between the embeddings of the drug mechanism and the embeddings of the genes generated by end-to-end neural networks. For drug i and cell line j , the detailed gene-selection steps are as follows.

First, the embedding of drug mechanism $\left(Z_{T}^{i}\right)$ is extracted by feeding the drug-specific heterogeneous network $\left(G^{i}\right)$ into a GNN module, composed of three layers of Graph Attention Network (Velickovic et al., 2017) with Top-k pooling layer (Gao and Ji, 2019; Cangea et al., 2018; Knyazev et al., 2019). Cell line gene expression values are used as the node features for each gene nodes whereas 0 is assigned to the pathway nodes, which can be formulated as Equation 1:

$$Z_{T}^{i}=\text{GNN}\left(G^{i},X_{C}^{j}\right),\;G^{i}=\left(V^{i},E^{i}\right)$$ (1)

$X_{C}^{j}\in R^{g}$ is the gene expression profile vector of cell line j , where g is the number of genes. $V^{i}$ and $E^{i}$ are the set of nodes and edges in the heterogeneous network for drug i respectively. Next, the genes in the cell line j are embedded into vectors $Z_{G}^{j}$ using a Multi-Layer Perceptron (MLP) as in Equation 2:

$$Z_{G}^{j}=\text{GeneENC}\left(X_{C}^{j}\right)$$ (2)

Then, the dot products between each gene embedding vector (each row) in the resulting gene embedding matrix $Z_{G}^{j}\in R^{g\times d}$ and the drug mechanism vector $Z_{T}^{i}\in R^{d}$ are calculated to obtain the similarity scores $\left(S^{\left(i,j\right)}\in R^{g}\right)$ between the mechanism of the drug and each gene as shown in Equation 3:

$$S^{\left(i,j\right)}=Z_{G}^{j}\cdot Z_{T}^{i}$$ (3)

The genes are subsequently ranked according to their similarity scores to construct a mask $m_{k}^{\left(i,j\right)}\in R^{g}$ . In this mask, positions corresponding to the top k scoring genes are assigned the value 1, while all other positions are assigned the value 0 as shown in Equation 4:

$$m_{k}^{\left(i,j\right)}=1\left(r_{k}\left(S^{\left(i,j\right)}\right)\in \text{top }k\text{ genes}\right)$$ (4)

where $r_{k}\left(\cdot \right)$ is the ranking operator that identifies top k similarity score genes, and 1(⋅) is the indicator function that takes on the value 1 if the gene belongs to the top k genes and 0 otherwise. The specific value for k used in this study is 100. Finally, the gene expression profile of the cell line is filtered by applying the calculated mask as shown in Equation 5:

$${X^{\prime }}_{C}^{\left(i,j\right)}=X_{C}^{j}*m_{k}^{\left(i,j\right)}.$$ (5)

Hence, our deep learning and embedding similarity-based re-ranking strategy ensures that the selected genes are not only relevant to the drug mechanism but also contextually interconnected, compared to the naïve network propagation score-based ranking, where each scores are independent. This approach enhances the utility of gene selection by providing a more biologically meaningful and context-aware set of genes.

### 2.4 Drug response prediction step

After acquiring the drug mechanism embedding and selecting the relevant genes, the filtered cell line gene expression profile, along with the drug property data, is fed into the predictor module, which then determines the final drug response values. Initially, the filtered cell line gene expression profiles ${X^{\prime }}_{C}$ and the drug structural information $X_{D}$ for drug i and cell line j are separately input into the cell line encoder and the drug encoder, respectively as shown in Equations 6, 7:

$$Z_{C}^{\left(i,j\right)}=\text{CellENC}\left({X^{\prime }}_{C}^{\left(i,j\right)}\right)$$ (6)

$$Z_{D}^{i}=\text{DrugENC}\left(X_{D}^{i}\right).$$ (7)

The cell line representation dynamically adapts to the drug input due to the drug-specific gene selection filters. During our experiments, we utilized the Extended-Connectivity Fingerprints (ECFP) with a dimension of 128 and a radius of 2 as the structural information for each drug, computed through the RDKit Python package.

Following the acquisition of fixed-size embedding vectors for both the cell line $\left(Z_{C}^{\left(i,j\right)}\in R^{d}\right)$ and the drug $\left(Z_{D}^{i}\in R^{d}\right)$ , these vectors are concatenated with the drug mechanism vector $Z_{T}^{i}$ from Equation 1. This combined vector is then input into the final fully connected layers of the predictor module, resulting in the output of the final predicted IC50 value ${\hat{y}}^{\left(i,j\right)}$ as shown in Equation 8:

$${\hat{y}}^{\left(i,j\right)}=\text{pred}\left(\left[Z_{D}^{i},\;Z_{C}^{\left(i,j\right)},\;Z_{T}^{i}\right]\right)$$ (8)

Here, [⋅,⋅] represents the concatenation operation. In essence, our model is designed to select genes in a way that is guided by domain knowledge. This method enables a more precise and informed selection, thereby enhancing the accuracy and reliability of our predictions.

The overall hyperparameter selection on the neural network parameter search space and network databases are detailed in the Supplementary Tables S1, S2, respectively.

### 2.5 Experimental setup

In the context of drug response prediction, each sample is a pair of a drug and a cell line. Traditional machine learning methods of splitting data into train, validation, and test sets could potentially lead to overestimating the performance of a model due to repeated exposure to the same drugs and cell lines that the model has already seen during training. To address this issue, we employed a data splitting strategy that completely blinds the model to certain drugs during training, which we refer to as “drug split”. In the “drug split” scenario, the test set comprises only those drugs that the model has not encountered during training. This method is crucial for assessing whether the model has effectively learned the general characteristics of drugs.

A common phenomenon observed in previous studies (Nguyen et al., 2021; Koras et al., 2021; Pak et al., 2023) is that prediction performance is much lower when models are tested on samples with unseen drugs during training compared to when tested on samples with unseen cell lines. Specifically, for data split settings based on cell lines, drug response prediction models exhibit Pearson correlation coefficient (PCC) performance of around 0.9, whereas in the drug split setting, models show average PCC values around 0.3 to 0.4. Given that model performance in the cell line split is saturated while there is significant room for improvement in the drug split, this study focuses on performance comparisons in the drug split setting.

We trained all the models for 100 epochs with early stopping patience of 5 epochs, applying a uniform learning rate of 1e-4. For robust performance measurement, each model training was carried out using 5-fold cross-validation. The details of the hyperparameter search space for DGDRP are described in Supplementary Table S1. All the models used the same random seeds, and the average performance metric values across all the seeds and cross-validations are reported as the final performance measurements.

#### 2.5.1 Evaluation metrics

For each experiment, we compared the predicted drug response values $\hat{y}$ with the actual ground truth IC50 values y using various metrics to ensure fair comparisons. The objective of the experiments is to verify if the drug response predictions of our model demonstrate a significant improvement over existing prediction methods. As drug response prediction fundamentally takes on the form of a regression task, we used traditional regression metrics such as Root Mean Square Error (RMSE) to quantify accuracy. Furthermore, we evaluated the performance using additional metrics such as Pearson Correlation Coefficient (PCC) and Spearman Correlation Coefficient (SCC), offering a diverse view on the prediction performance of the models. Each metric was calculated as shown in Equations 9–11:

$$RMSE\left(y,\hat{y}\right)=\sqrt{\frac{\sum_{i=1}^{n}{\left(y_{i}-{\hat{y}}_{i}\right)}^{2}}{n}}$$ (9)

$$PCC\left(y,\hat{y}\right)=\frac{\sum_{i=1}^{n}{\left(y_{i}-{\hat{y}}_{i}\right)}^{2}}{\sigma_{y_{i}}\sigma_{{\hat{y}}_{i}}}$$ (10)

$$SCC\left(y,\hat{y}\right)=PCC\left(r\left(y\right),r\left(\hat{y}\right)\right)$$ (11)

where r (y) and r $\left(\hat{y}\right)$ denote the ranked vectors of y and $\hat{y}$ , respectively.

These metrics are computed based on the difference between the actual drug response values $\left(y\in R^{n}\right)$ and the predicted drug response values $\left(\hat{y}\in R^{n}\right)$ , where n represents the number of samples consisting of drug and cell line pairs. $y_{i}$ and $\hat{y_{i}}$ denote the drug response value of the i th sample.

## 3 Results

The goal of DGDRP is to select genes in drug-specific and adaptive way so that the gene selection process can be guided by domain knowledge and also be integrated into the learning process in the hope that such method can enhance the performance of drug response prediction. In this section, we show how integrating heterogeneous network built based on drug target information contributes to improving drug response prediction by evaluating the performance of the proposed method.

### 3.1 Drug response prediction performance comparison

To demonstrate and assess the effectiveness of our framework as a stand-alone drug response prediction model, we compared the performance of DGDRP with other state-of-the-art (SOTA) deep learning-based models on the GDSC dataset. For this experiment, DGDRP was compared to six other models: Adaptive Gene Weighting (AGW), DEERS (Koras et al., 2021), DeepTTA (Jiang et al., 2022), Precily (Chawla et al., 2022), GPDRP (Yang and Li, 2023), and a baseline MLP. The AGW model draws inspiration from the SRDFM (Su et al., 2022) model, with the “Outcome Generation Component” replaced by an MLP, as SRDFM outputs the rank of drug response instead of the actual drug response value.

Table 1 presents the performance comparison results. DGDRP achieved the highest Pearson correlation coefficient (PCC) and the lowest root mean square error (RMSE), and it secured the second-best Spearman correlation coefficient (SCC). These results indicate that DGDRP delivers SOTA performance as an independent drug response prediction model, even without pre-filtering the input gene expression data, unlike some of the other models in comparison.

**TABLE 1:** Drug response prediction performance comparison against SOTA deep learning models on GDSC dataset under drug-split. The best performance is highlighted in bold, and the second-best performance is underlined. The standard deviation is indicated as ± .

Prediction models	PCC (↑)	RMSE (↓)	SCC (↑)	Model description
**DGDRP** (ours)	**0.5154** ( ± 0.045)	**2.3180** ( ± 0.083)	0.4140 ( ± 0.063)	Network propagation and GNN-based model
GPDRP (Yang and Li, 2023)	0.4730 ( ± 0.076)	2.4045 ( ± 0.273)	0.4015 ( ± 0.058)	Pathway activity score-based Graph Transformer model
Precily (Chawla et al., 2022)	0.4673 ( ± 0.125)	2.7150 ( ± 0.240)	**0.4192** ( ± 0.134)	Pathway-based deep neural network
DeepTTA (Jiang et al., 2022)	0.4241 ( ± 0.155)	2.5096 ( ± 0.358)	0.3771 ( ± 0.117)	Transformer-based model
AGW (Su et al., 2022)	0.3683 ( ± 0.149)	2.6053 ( ± 0.297)	0.3373 ( ± 0.146)	Siamese neural network-based model
DEERS (Koras et al., 2021)	0.2939 ( ± 0.132)	2.6225 ( ± 0.375)	0.2743 ( ± 0.108)	Auto-Encoder-based model
MLP	0.3799 ( ± 0.129)	2.5871 ( ± 0.292)	0.3433 ( ± 0.120)	Baseline model

### 3.2 Gene-selection methods comparison

In order to quantitatively evaluate the effectiveness of the proposed gene-selection method, we first directly compared the performance against four different gene-selection settings, namely, the selection of “genes with high variance”, “landmark genes from LINCS L1000”, “genes selected by Machine Learning (ML)-driven feature selection method”, and “all genes (10,000 genes)”. For “genes selected by data-driven feature selection method”, we obtained the genes using the L1 regularization-based feature selection method using the Scikit-learn Python package (Pedregosa et al., 2011). The architectures of the predictor modules (fully-connected layers) are the same for all models being compared, including the DGDRP.

As shown in Table 2, DGDRP shows the best PCC and RMSE performance. For SCC performance, DGDRP shows the second best value. Considering the fact that the model for the best performing method “ML-driven (L1)” was pre-exposed to the samples and the corresponding drug response values during gene-selection phase, our model still achieved a comparable performance even without such advantage. Moreover, only the proposed method has the characteristics of both drug-specificity and the ability to be run in end-to-end fashion.

**TABLE 2:** Gene selection methods comparison in drug-split on two databases: GDSC and NCI-60. The best performance is highlighted in bold, and the second-best performance is underlined. The standard deviation is indicated as ± .

Selection Methods	Drug-specific	End-to-end	GDSC		NCI-60	
			PCC (↑)	RMSE (↓)	PCC (↑)	RMSE (↓)
**DGDRP**	Yes	Yes	**0.5154** ( ± 0.045)	**2.3180** ( ± 0.083)	**0.4390** ( ± 0.02)	0.8431 ( ± 0.01)
ML-Driven (L1)	Yes	No	0.4621 ( ± 0.058)	2.4020 (± 0.225)	0.3537 ( ± 0.30)	**0.8155** ( ± 0.08)
High Variance	No	No	0.3849 ( ± 0.075)	2.4522 ( ± 0.218)	0.3464 ( ± 0.01)	0.8551 ( ± 0.01)
Landmark Genes	No	No	0.3820 ( ± 0.072)	2.4408 ( ± 0.254)	0.3385 ( ± 0.02)	0.8557 ( ± 0.01)
All Genes (10,000)	No	No	0.3655 ( ± 0.076)	2.5048 ( ± 0.278)	0.3261 ( ± 0.01)	0.8589 ( ± 0.00)

### 3.3 Ranking and re-ranking approach enables accurate predictions

Next, an ablation study was conducted to investigate the contribution of each element within the heterogeneous network towards the prediction of drug response. As described in Section 2.3.2, the heterogeneous network comprises a unique structure for each drug encompassing its direct target genes, the indirect target genes derived via the NetGP algorithm, and the pathways containing these target genes. In this section, a comparison has been made among the heterogeneous networks of the following structures: network inclusive of all direct targets, indirect targets, and pathway nodes; network without pathway nodes; and network without both indirect targets and pathway nodes. For network without both indirect targets and pathway nodes, the topology was obtained from the STRING PPI network. To reduce the size of the network to match the size of the original heterogeneous network, the PPI network was filtered to contain only the edges with STRING combined score of over 990 while preserving any edges that incorporated a target gene.

Table 3 shows that the heterogeneous networks that contain all direct targets, indirect targets, and pathway nodes achieved the best performance in all three metrics. Interestingly, the networks without both indirect targets and pathway nodes showed better performance than the networks without pathway nodes but with indirect targets. It can be hypothesized that indirect targets and pathways work in a combinatorial way to learn the characteristics of drugs.

**TABLE 3:** Ablation study across different heterogeneous network structures. The best performance is highlighted in bold, and the second-best performance is underlined. The standard deviation is indicated as ± .

Network structure	PCC (↑)	RMSE (↓)	SCC (↑)
**DGDRP**	**0.5154 ( ± 0.045)**	**2.3180 ( ± 0.083)**	**0.4140 ( ± 0.063)**
DGDRP w/o Pathway	0.4939 ( ± 0.063)	2.3656 ( ± 0.118)	0.3994 ( ± 0.069)
DGDRP w/o (Pathway, Indirect targets)	0.5034 ( ± 0.065)	2.3469 ( ± 0.118)	0.4054 ( ± 0.072)

### 3.4 Investigation on selected gene sets and drugs’ mechanism of action

The proposed method of gene selection can also bring interpretability to the deep learning model. Using the top k score genes (Figure 1), gene set for each drug-cell line pair can be obtained. To investigate the genes selected for a specific drug, the gene selection masks were extracted by running the model on the test set. The results were then filtered for each drug. The selected gene set was then used to conduct pathway enrichment analysis, allowing us to understand the biological mechanisms related to the selected genes. This allows us to compare the alignment of the selected genes with the MoA of the input drugs. The enrichment was performed on the pathways obtained from the KEGG pathway database (Kanehisa and Goto, 2000) using the gseapy (Subramanian et al., 2005; Mootha et al., 2003; Xie et al., 2021) python package, with enrichment background genes set as STRING PPI genes.

The results show that the selected genes enrich pathways related to the MoA of the drugs with statistical significance (Adjusted *p*-value < 0.05).

**FIGURE 3:**
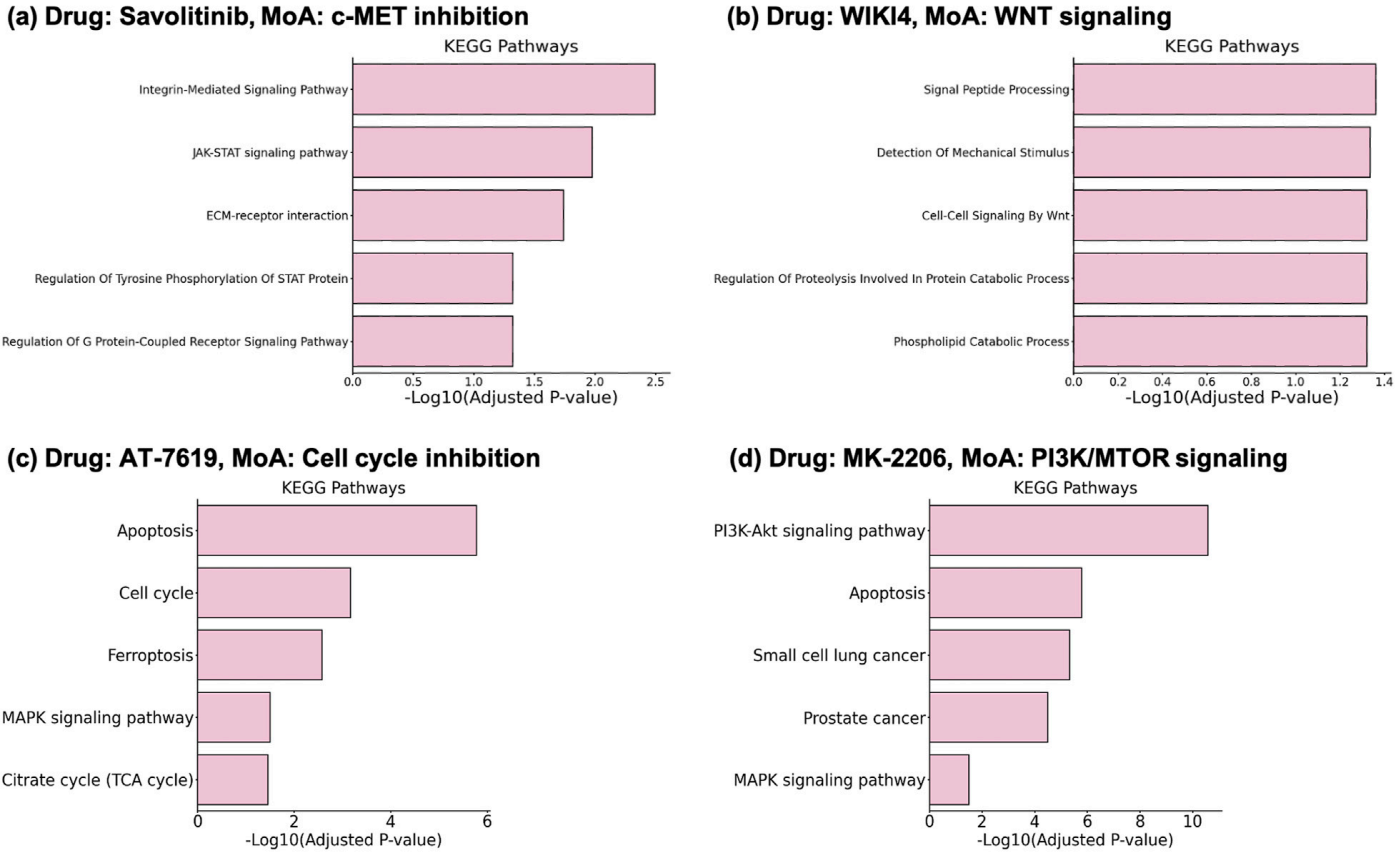
Enrichment results on the selected gene sets from DGDRP. Related pathways significantly enriched for **(A)** Savolitinib, **(B)** WIKI4, **(C)** AT-7519, and **(D)** MK-2206 are well aligned with the MoA of the drugs.

Savolitinib (Figure 3A) is an anti-cancer drug with MoA of c-MET (Hepatocyte growth factor receptor) inhibition (Markham, 2021). c-MET inhibition directly affects signaling mediated by the phosphorylation of STAT proteins, which are associated with the JAK-STAT pathway. This leads to the enrichment of terms such as “JAK-STAT signaling pathway” and “Regulation Of Tyrosine Phosphorylation Of STAT Protein”. The c-Met-integrin cooperation or c-Met/ β 1 Integrin Complex is a well-studied interaction (Henry et al., 2016), correlating with the “Integrin-Mediated Signaling Pathway” term. Additionally, c-MET activation through GPCRs Barrow-McGee et al. (2016) provides a clue for the enrichment of the “Regulation Of G Protein-Coupled Receptor Signaling Pathway” term.

WIKI4 is a potent inhibitor of TNKS2 (tankyrase), a component of the Wnt/ β -catenin signaling pathway (James et al., 2012) (Figure 3B). WIKI4 inhibition leads to decreased expression of β -catenin target genes and affects cellular responses to Wnt/ β -catenin signaling, which aligns with the “Cell-Cell Signaling By Wnt” term listed among the top-5 enriched terms. Wnt signaling is known to regulate cellular protein catabolism (Albrecht et al., 2019), which is further associated with the “Regulation Of Proteolysis Involved In Protein Catabolic Process” and “Signal Peptide Processing” terms.

Compound AT-7519 is a drug known to target “Cell cycle” according to GDSC and DrugBank (Yang et al., 2012; Wishart et al., 2018). The enrichment analysis result (Figure 3C) shows that indeed Cell cycle and related pathways such as Apoptosis are significantly enriched. In addition, the result for MK-2206 which targets “PI3K/MTOR signaling” also shows PI3K-Akt signaling pathway as one of the most significantly enriched pathways (Figure 3D).

Such results suggest that DGDRP was able to take biological mechanisms of the drugs into consideration when performing gene-selection in an end-to-end manner.

## 4 Discussion

In this study, we developed DGDRP, a drug-specific and adaptive gene-selection model for drug response prediction, leveraging a heterogeneous network constructed from drug target information and protein-protein interaction (PPI) networks. Unlike most existing studies that use cell data with genes as features, DGDRP addresses the high-dimension and low-sample problem inherent in drug response prediction. Typically, the number of genes far exceeds the number of available samples, necessitating dimensionality reduction through gene selection.

Traditional methods reduce gene numbers using pre-defined gene sets, which do not consider the unique characteristics of each drug and cannot be integrated into the learning process for end-to-end prediction. To overcome these limitations, we introduced a novel rank-and-re-rank computational approach that adaptively selects genes guided by domain knowledge. Since drugs exert their effects by perturbing target proteins, and these perturbations propagate through the cell via protein-protein interactions, we constructed a heterogeneous network comprising target proteins and related pathways for each drug.

Our approach utilizes embeddings extracted from this heterogeneous network along with cell line gene data to select genes based on similarity scores between the network and the genes. This adaptive selection process ensures that the chosen genes are biologically relevant to the drug’s mechanism of action.

The DGDRP model outperforms existing gene selection methods by offering a more precise and informed selection of genes, leading to improved prediction accuracy. Additionally, DGDRP demonstrates state-of-the-art performance as an independent drug response prediction model, achieving superior results without requiring pre-filtering of input gene expression data. This highlights the robustness and efficacy of our rank-and-re-rank approach in integrating both knowledge and data for more accurate drug response predictions.

Our proposed model poses two limitations. First, DGDRP relies on the availability of known drug target information for constructing the drug mechanism network. This dependency limits the model’s applicability to drugs with well-characterized targets. For novel drugs or those with unknown targets, DGDRP cannot be directly applied. One potential solution is to use drug-target interaction models to infer potential targets for these drugs, which can then be integrated into the DGDRP framework. Second, the quality and completeness of the biological networks (e.g., PPI networks) used in DGDRP can introduce biases. Incomplete or biased network data may lead to the exclusion of relevant genes or the inclusion of irrelevant ones, affecting the accuracy of gene selection and drug response prediction. Hence, continuous updates and validation of the biological networks are essential. Incorporating multiple network sources and cross-validating results can help mitigate this bias.

Overall, DGDRP represents a significant advancement in the field of drug response prediction by offering a robust gene selection framework that integrates both domain knowledge and data-driven approaches, enhancing prediction accuracy and enabling effective biomarker discovery, simultaneously.

## Data Availability

The original contributions presented in the study are included in the article/Supplementary Material, further inquiries can be directed to the corresponding author.
