# Supplementary material for: DGDRP: drug-specific gene selection for drug response prediction via re-ranking through propagating and learning biological network
Source: Front Genet. 2024 Sep 20;15:1441558. doi: 10.3389/fgene.2024.1441558 (PMC11450864; doi:10.3389/fgene.2024.1441558)
Supplement: Supplementary file 1 [file DataSheet1.PDF]

DGDRP: Drug-specific Gene selection for Drug Response Prediction via re-ranking through propagating and learning biological network

1 SUPPLEMENTARY TABLES AND FIGURES

1.1 Supplementary Tables

Table S1. Hyperparameter search space for DGDRP.

Hyperparameter	Search space	Selected parameter
Batch_size	[16, 32, 64]	64
Learning rate	[1e-4, 5e-4, 1e-3, 5e-3]	0.0001
Weight decay	[0, 1e-6, 1e-5, 1e-4]	0.000001
GNN type	[GAT, GCN] + BatchNorm + TopKPooling	GAT + BatchNorm + TopKPooling
GNN #layers	[2, 3]	3
GNN hid dim	[256, 512]	512
Cell/Drug embed #layers	[2, 3]	2
Cell/Drug embed hid dim	[128, 256]	128
Predictor #layers	2e	2
Predictor hid dim	128	128
Predictor dropout	[0.2, 0.5, 0.6]	0.6
Top-k for ranking and drug mechanism network construction	[20, 25, 50]	20
Top-k for re-ranking with masking	[100, 500, 1,000]	1000

Table S2. Performance of DGDRP models across different STRING score thresholds and pathway types (NCI-60 dataset). The best performance is highlighted in bold, and the second-best performance is underlined. The standard deviation is indicated as ±.

Pathway Type	STRING Cutoff	PCC (↑)	SCC (↑)	RMSE (↓)
KEGG	0.9	**0.4390** (± 0.02)	**0.4487** (± 0.02)	**0.8431** (± 0.01)
Reactome	0.9	<u>0.4324</u> (± 0.02)	<u>0.4404</u> (± 0.03)	<u>0.8464</u> (± 0.01)
KEGG	0.7	0.4155 (± 0.02)	0.4262 (± 0.02)	0.8588 (± 0.01)
Reactome	0.7	0.4182 (± 0.02)	0.4254 (± 0.02)	0.8532 (± 0.01)

Table S3. Performance of DGDRP models across different PPI networks (GDSC dataset). The best performance is highlighted in bold, and the second-best performance is underlined. The standard deviation is indicated as ±.

PPI Network Type	PCC (↑)	SCC (↑)	RMSE (↓)
STRING	**0.5154** (± 0.045)	**0.4140** (± 0.0063)	**2.3180** (± 0.083)
HitPredict	<u>0.4305</u> (± 0.072)	<u>0.3616</u> (± 0.075)	<u>2.5271</u> (± 0.117)

Table S4. Statistics of pathway databases KEGG and Reactome utilized for drug mechanism network construction.

Database	The number of terms	Gene coverage	Genes per Term
KEGG	320	8,078	102
Reactome	1,818	10,489	61
